# Obesidade e Gordura Epicárdica Associados à Maior Recorrência de Fibrilação Atrial após Ablação: Apenas Coincidência?

**DOI:** 10.36660/abc.20220103

**Published:** 2022-04-07

**Authors:** Cristiano F. Pisani, Mauricio Scanavacca

**Affiliations:** 1 InCor FMUSP São Paulo SP Brasil Unidade Clínica de Arritmia do Instituto do Coração (InCor) do Hospital das Clínicas da FM USP (HC-FMUSP), São Paulo, SP – Brasil

**Keywords:** Fibrilação Atrial, Obesidade, Ablação por Cateter

A fibrilação atrial (FA) é a arritmia sustentada mais comum na prática clínica e a sua prevalência aumenta com a idade.^1^É uma condição decorrente de múltiplos aspectos fisiopatológicos, não exclusivamente eletrofisiológicos, provenientes de extrassístoles deflagradas a partir das veias pulmonares. Associa-se a diferentes doenças cardiovasculares e sistêmicas, sendo a obesidade um dos fatores de risco já comprovadamente conhecido.

Indivíduos com sobrepeso e obesos apresentam risco maior para ocorrência de FA. Para cada unidade de incremento do IMC, o risco corrigido para incidência de FA aumenta de 3 a 7% em relação a indivíduos com IMC abaixo de 25.^2,3^ Os mecanismos que justificam essa observação não são completamente entendidos e, provavelmente, multifatoriais. Pacientes obesos frequentemente apresentam hipertensão arterial^4^ e apneia obstrutiva do sono associadas, fatores já considerados como predisponentes à FA.^5^ Um estudo recente demonstrou que a implementação de um programa de controle de peso tem impacto importante no controle clínico desta arritmia.^6^

Thanassoulis e colaboradores ressaltaram uma associação entre a extensão da gordura epicárdica e maior ocorrência de FA (odds ratio = 1,28 por desvio padrão de volume de gordura epicárdica), porém essa observação não se aplica à quantidade de gordura intratorácica e abdominal.^7^ Portanto, essa associação não pode ser considerada exclusivamente pela existência de obesidade e suas consequências. Uma das justificativas é a inflamação atrial induzida pela gordura epicárdica. A FA é uma arritmia associada ao aumento de marcadores inflamatórios, como proteína C reativa, interleucinas e fator de necrose tumoral.^8^ Essas substâncias podem ser produzidas pelo tecido adiposo atrial, causando inflamação no miocárdio adjacente. A gordura epicárdica contém níveis elevados de células inflamatórias em comparação com a gordura subcutânea, incluindo mastócitos, macrófagos e linfócitos.^9,10^

Em um estudo experimental 10 ovelhas alimentadas com dieta hipercalórica por 36 semanas foram comparadas com um grupo controle, com alimentação convencional. Os animais foram submetidos a estudo eletrofisiológico e mapeamento eletroanatômico do átrio esquerdo. As ovelhas obesas apresentaram maior volume e pressão do AE, maior heterogeneidade da condução elétrica, aumento na quantidade de eletrogramas atriais fracionados, redução na voltagem dos eletrogramas na parede posterior do AE e maior heterogeneidade de voltagem, sugerindo a presença de um substrato fibrótico atrial como possível determinante dessa associação entre obesidade e FA.^11^

Nessa edição dos Arquivos Brasileiros de Cardiologia, Matos et al.,^12^ estudaram uma série de 68 pacientes que haviam sido submetidos a angiotomografia de coronária, ressonância magnética cardíaca com realce 3D (utilizando software ADAS 3D - Galgo Medical) antes de um primeiro procedimento de ablação de fibrilação atrial. Observaram uma fraca, porém estatisticamente significativa correlação entre fibrose atrial no mapeamento eletroanatômico e tecido adiposo epicárdico medido na tomografia em um corte ao nível do tronco da coronária esquerda (TAE_TC_) - coeficiente de correlação de Spearman = 0,40, p=0,001.

Os pacientes foram seguidos por um período mediano de 22 meses (IIQ 12-31), observando-se recorrência de FA em 31 pacientes (46%). Os que apresentaram recorrência eram mais propensos a apresentar FA não paroxística, tinham maior volume atrial e maiores volumes de tecido adiposo e fibrose atrial. Na análise multivariada, no entanto, apenas a TAE_TC_ e FA não paroxística associaram-se a maior recorrência. Essa observação é interessante e difere de estudos prévios,^13-15^ nos quais a recorrência relacionou-se à extensão da fibrose atrial.

Esses achados corroboram a importância da gordura epicárdica não apenas na geração de fibrose, mas também como fator inflamatório. Ainda, influências autonômicas poderiam contribuir para maior recorrência da arritmia, pois na gordura epicárdica estão localizados os plexos ganglionares que, inclusive, podem ser alvos na ablação da FA.^16-18^

Apesar do crescente entendimento dos mecanismos fisiopatológicos envolvidos na ocorrência de FA, e constante aprimoramento técnico dos procedimentos de ablação por cateter, há muitos aspectos ainda não bem esclarecidos. Entre eles, sua associação com obesidade e extensão da gordura epicárdica atrial.
