# Supplementary material for: Obesidade e Gordura Epicárdica Associados à Maior Recorrência de Fibrilação Atrial após Ablação: Apenas Coincidência?
Source: Arq Bras Cardiol. 2022 Apr 7;118(4):743–4. [Article in Portuguese] doi: 10.36660/abc.20220103 (PMC9007023; doi:10.36660/abc.20220103)
Supplement: Translation

# Translation: Obesity and Epicardial Fat Associated with Higher Atrial Fibrillation Recurrence After Ablation: Just Coincidence?

**Authors:** Cristiano F. Pisani, Mauricio Scanavacca

**Affiliations:** 1 Brazil Unidade Clínica de Arritmia do Instituto do Coração (InCor) do Hospital das Clínicas da FM USP (HC-FMUSP), São Paulo, SP – Brazil

**Keywords:** Atrial Fibrillation, Obesity, Catheter Ablation

Atrial fibrillation (AF) is the most common sustained arrhythmia in clinical practice, and its prevalence increases with age.^1^ It is a condition resulting from multiple pathophysiological aspects, not exclusively electrophysiological, from extrasystoles triggered by the pulmonary veins. It is associated with different cardiovascular and systemic diseases, obesity being a known risk factor.

Overweight and obese individuals are at greater risk for the occurrence of AF. For each unit of increment in BMI, the corrected risk for incidence of AF increases from 3 to 7% concerning individuals with a BMI below 25.^2,3^ The mechanisms that justify this observation are not fully understood and are probably multifactorial. Obese patients often have arterial hypertension^4^ and associated obstructive sleep apnea, factors already considered predisposing to AF.^5^ A recent study demonstrated that implementing a weight control program has an important impact on the clinical control of this arrhythmia.^6^

Thanassoulis et al. highlighted an association between the extent of epicardial fat and greater occurrence of AF (odds ratio = 1.28 per standard deviation of epicardial fat volume). However, this observation does not apply to the amount of intrathoracic and abdominal fat.^7^ Therefore, this association cannot be considered exclusively by the existence of obesity and its consequences. One of the justifications is the atrial inflammation induced by epicardial fat. AF is an arrhythmia associated with increased inflammatory markers, such as C-reactive protein, interleukins and tumor necrosis factor.^8^ These substances can be produced by the atrial adipose tissue, causing inflammation in the adjacent myocardium. Epicardial fat contains higher inflammatory cells levels than subcutaneous fat, including mast cells, macrophages, and lymphocytes.^9^

In an experimental study, 10 sheep fed a hypercaloric diet for 36 weeks were compared with a control group with conventional feeding. The animals were submitted to an electrophysiological study and electroanatomical mapping of the left atrium. Obese sheep presented greater LA volume and pressure, greater electrical conduction heterogeneity, increased amount of fractionated atrial electrograms, reduced voltage of electrograms in the posterior LA wall and greater voltage heterogeneity, suggesting the presence of an atrial fibrotic substrate such as a possible determinant of this association between obesity and AF.^11^

In this issue of *Arquivos Brasileiros de Cardiologia*, Matos et al.,^12^ studied 68 patients who had undergone coronary CT angiography, cardiac magnetic resonance imaging with 3D enhancement (using ADAS 3D software - Galgo Medical) before a first ablation procedure of atrial fibrillation. They observed a weak but statistically significant correlation between atrial fibrosis on electroanatomical mapping and epicardial adipose tissue measured on tomography in a slice at the left main coronary artery (TAE_TC_) - Spearman correlation coefficient = 0.40, p=0.001.

Patients were followed up for a median period of 22 months (IQI 12-31), with AF recurrence in 31 patients (46%). Those who had recurrence were more likely to have non-paroxysmal AF, had larger atrial volume and larger volumes of adipose tissue and atrial fibrosis. However, in the multivariate analysis, only TAE_TC_ and non-paroxysmal AF were associated with greater recurrence. This observation is interesting and differs from previous studies,^13-15^ in which recurrence was related to the extent of atrial fibrosis.

These findings corroborate the importance of epicardial fat not only in the generation of fibrosis but also as an inflammatory factor. Furthermore, autonomic influences could contribute to a greater arrhythmia recurrence, as the ganglion plexuses are located in the epicardial fat, which can even be targeted in AF ablation.^16-18^

Despite the growing understanding of the pathophysiological mechanisms involved in the occurrence of AF and the constant technical improvement of catheter ablation procedures, many aspects are still not well understood. Among them is its association with obesity and atrial epicardial fat extension.
